# Comparative review of three cost-effectiveness models for rotavirus vaccines in national immunization programs; a generic approach applied to various regions in the world

**DOI:** 10.1186/1741-7015-9-84

**Published:** 2011-07-08

**Authors:** Maarten J Postma, Mark Jit, Mark H Rozenbaum, Baudouin Standaert, Hong-Anh Tu, Raymond CW Hutubessy

**Affiliations:** 1 Unit of PharmacoEpidemiology & PharmacoEconomics (PE2), Department of Pharmacy, University of Groningen, Antonius Deusinglaan 1, 9713 AV, Groningen, the Netherlands; 2 Modelling and Economics Unit, Health Protection Agency (HPA), 61 Colindale Avenue, London NW9 5EQ, UK; 3 Health Economics Department, GlaxoSmithKline Biologicals (GSKBio), Rue de l'Institut 89 1330 Rixensart, Wavre, Belgium; 4 Initiative for Vaccine Research, World Health Organisation (WHO), Avenue Appia, 1211 Geneva 27, Switzerland

## Abstract

**Background:**

This study aims to critically review available cost-effectiveness models for rotavirus vaccination, compare their designs using a standardized approach and compare similarities and differences in cost-effectiveness outcomes using a uniform set of input parameters.

**Methods:**

We identified various models used to estimate the cost-effectiveness of rotavirus vaccination. From these, results using a standardized dataset for four regions in the world could be obtained for three specific applications.

**Results:**

Despite differences in the approaches and individual constituting elements including costs, QALYs Quality Adjusted Life Years and deaths, cost-effectiveness results of the models were quite similar. Differences between the models on the individual components of cost-effectiveness could be related to some specific features of the respective models. Sensitivity analysis revealed that cost-effectiveness of rotavirus vaccination is highly sensitive to vaccine prices, rotavirus-associated mortality and discount rates, in particular that for QALYs.

**Conclusions:**

The comparative approach followed here is helpful in understanding the various models selected and will thus benefit (low-income) countries in designing their own cost-effectiveness analyses using new or adapted existing models. Potential users of the models in low and middle income countries need to consider results from existing studies and reviews. There will be a need for contextualization including the use of country specific data inputs. However, given that the underlying biological and epidemiological mechanisms do not change between countries, users are likely to be able to adapt existing model designs rather than developing completely new approaches. Also, the communication established between the individual researchers involved in the three models is helpful in the further development of these individual models. Therefore, we recommend that this kind of comparative study be extended to other areas of vaccination and even other infectious disease interventions.

## Background

Various countries are currently in the process of evaluating whether or not to include rotavirus vaccination in their national immunization programs. Two rotavirus vaccines are currently available: Rotarix^® ^and Rotateq™, marketed by GlaxoSmithKline (GSK) and Sanofi Pasteur MSD (SPMSD), respectively. Rotavirus vaccines have proven to be efficacious in preventing rotavirus-related disease, gastrointestinal disease and health-care use (general practitioner (GP) visits and hospitalizations) in infants and toddlers [1-3].

Health-economic properties of these new vaccines present one characteristic to be analyzed with respect to their inclusion in National Immunization Programs (NIPs). Numerous health-economic analyses already exist regarding rotavirus vaccination, mostly in high-income country settings, some being single-country analyses but a few multi-country analyses [4-6]. Also, one critical review of these existing models has already been performed [7]. Recently, some studies have become available targeted at situations in low- and middle-income countries, involving all regions of the world (for example, Vietnam, China, Kenya and Colombia [8-11]). A review of these studies in Asia, Africa and South America is in preparation [12]. Based on the favorable cost-effectiveness profile of many of the models applied in low- and middle-income countries, the World Health Organization (WHO) advises inclusion of rotavirus vaccines in the NIPs [13]. However, in general, it is still not always clear which aspects these individual models differ on, what impact such differences have on the cost-effectiveness outcomes and which model might be preferred over the others in continental, regional or country specific situations [14].

This study aims to critically review some of these available cost-effectiveness models for rotavirus vaccination, compare their designs using a standardized approach and compare similarities and differences in cost effectiveness outcomes using a uniform set of input parameters. WHO has initiated this study to enable providing guidance to low- and middle-income countries if requested, on the strengths and weaknesses of existing health-economic models for rotavirus vaccination as a basis for decisions about whether or not to build their own models or to adapt existing models to local situations. In particular, our study intends to help WHO to guide individual countries on rotavirus modelling, specifically national decision makers who may have the interest, research capacity and resources to conduct their own cost-effectiveness analyses in generating evidence for decision making on whether or not to introduce rotavirus vaccination. Therefore, our analysis might be most suited for lower middle income countries that have some capacity to attract global partners for model adaptation, whereas most low income countries may not have sufficient technical capacity for building or adapting existing models. Notably, our goal is neither to advocate for the use of specific models nor to recommend individual modelling groups over others.

## Methods

### Review of models

Initially, we searched for existing health-economic models in the literature using PubMed, Embase and Web of Science. Although the search was not limited to the English language, the relevant papers that emerged from the initial search and crude selection were all in English. A further selection was made for those models to be included in the detailed comparison based on various criteria related to our goals of achieving diversity in terms of provenance (public versus private), methods (single-cohort models, multi-cohort models and so on) and specific vaccines to be incorporated by the model (that is, two individual vaccines exist that are slightly different). Modelling groups were selected and contacted, explicitly based on these criteria. However, availability, ease of access, complexity and time investment of the individual research groups appeared to be the strongest criteria in practice for inclusion in the final comparison. In particular for those models with a high degree of complexity, the time investment required appeared to prevent the groups from becoming involved in our comparison. Also, for these complex models resource requirements for this analysis presented a strong limitation, which could not easily be overcome given the limited funds available by WHO for the endeavor.

### Comparative framework

As described, various cost-effectiveness models were identified by the process described above. Their developers were contacted by a WHO officer (RH) to invite them to participate in the model comparison. Subsequently, the model inclusion was co-ordinated by two authors (RH and MP), while one author (MP) included his own group's model into the comparison. The process resulted in three models provided to us, including analyses using the standardized dataset specified below (Table 1). From all modelling groups at least one co-author is included in the author list of this paper. These models represented a balanced public-private mix involving one designed by the pharmaceutical industry (Roxanne **Ro**tari**x**™ **An**alyses of **E**conomics from GSK), one developed by public financing within a European-Union project (POLYMOD) and one privately financed (Sanofi Pasteur MSD) but developed by the University of Groningen within the context of an unrestricted grant (CoRoVa **Co**nsensus **Ro**tavirus model **Va**ccination). Models included could be applied to modelling use of either vaccine (Rotarix or RotaTeq), hence ensuring that the unique features of the two vaccines were adequately represented in our analysis. In addition, both manufacturers were directly (Roxanne) or indirectly (CoRoVa) involved in the models. Only static models were compared; this posed both a disadvantage and an advantage. In particular, comparison with a dynamic model, which could explicitly analyze the effect of vaccination on the spread of rotavirus infection using mathematical modelling, would be extremely valuable. However, our selection of only static models for comparison enhances comparability of the individual models and facilitates understanding the differences that still remain between the models' outcomes.

**Table 1:** Basic characteristics of the models investigated^a^

	POLYMOD	Roxanne	CoRoVa
Developers	HPA	GSK	University Groningen
Funding	EU	GSK	SPMSD
Software platform	Excel	Excel	Excel
Dynamic vs. static	Static	Statistic	Static
Deterministic vs. stochastic	Deterministic	Deterministic	Deterministic
Open vs. closed	Open	Closed	Closed
Cohort vs. population-based	Multi-cohort	Cohort	Cohort

Special features	Stepwise waning	Breastfeeding effects modeled	In- between dose efficacies modeled

^a^Specific questions regarding the three models should be attended to Dr Raymond Hutubessy (email address: hutubessyr@who.int)

The owners of the models provided access to their models through physical transfer of the software - accompanied with user guides and/or publications - and explained various concepts and characteristics of these models face-to-face, through e-mail contacts and telephone calls during 2009. Additionally, during a one-day consultation in 2009 at the WHO headquarters in Geneva, concepts and draft results were discussed with a large group of experts in epidemiology, immunology, vaccinology, political sciences and health economics. In the end, final calculations were performed by the modellers themselves using the most current model versions at the end of 2010 and extensively discussed during the winter of 2010/2011. To summarize the way the three models were included in this analysis, we note that all the software for the models was physically available to the coordinating team (MP and RH), at least two face-to-face meetings between member(s) of the coordinating team and each research group were organized and final calculations were checked by the coordinating team and cross-checked for face validity by all three individual research groups.

The standardized approach in comparing these models involved stepwise analysis of the structure, the input parameters required and specific assumptions underlying the models.

### Details on available models

As mentioned, during the model selection process three models with corresponding results for the standardized input parameters became available to us. These were the POLYMOD model [4,15,16], Roxanne [17-20], and CoRoVa [21-23].

The POLYMOD-model was developed in the context of a European Union (EU)-funded project with the same acronym [4]. RVGE Rotavirus GastroEnteritis was modelled using an age-structured cohort model that followed cohorts of vaccinated and unvaccinated individuals (Table 1). For the first year of life, the cohort was stratified into monthly age groups, with one-year age bands applied beyond (one to five years old). The model was initially a single-cohort model; however, it was adapted to a multi-cohort model for the purpose of this exercise to allow a step-up in vaccination coverage. RVGE was stratified into mild disease with home treatment only, moderate disease with primary care visits (GP and/or hospital outpatient), severe disease with hospital emergency room (ER)-visits and/or admissions, nosocomial infections and death. QALY losses were incurred both by the index infants and their care givers. QALYs were taken from one specific study on the topic [23], which were quite comparable to those used in some other health-economic studies [5]. The model was designed by modellers based in England's Health Protection Agency (HPA) with input from modellers based in public health institutions in Belgium, Finland, France and the Netherlands and applied to model rotavirus epidemiology and cost-effectiveness of vaccination in the five countries of Belgium, England and Wales, Finland, France and the Netherlands. Unlike most static rotavirus models, waning vaccine immunity was explicitly incorporated into the model structure. Further country-specific models - based on this multi-cohort, multi-country model - have been published, for example, for the Netherlands [16].

Roxanne was developed as a Markov cohort process tree [17]. It is programmed in Microsoft Excel 2007 using Visual Basic and contains both cost-effectiveness and budget impact modules. The model was initially parameterized with data from France [18], but allows data from any country to be used [19]. A precursor of the model has been used to estimate the cost-effectiveness of Rotarix^® ^in the Netherlands [20]. Besides a comparison of vaccination versus no vaccination, the model was designed to additionally allow explicit comparison of two- and three-dose vaccination strategies. Obviously, outcomes in such analyses crucially depend on the exact characteristics and properties applied to the two- and three-dose vaccination schedules. Finally, Roxanne allows extensive probabilistic sensitivity analysis, using the At Risk add-in for Excel. A special feature of Roxanne involves the explicit inclusion of the modelling of maternal protection from (severe) infection through breastfeeding.

CoRoVa was initially developed for the specific Dutch situation and aimed at achieving consensus among various Dutch modelling groups that had previously worked on the cost-effectiveness of rotavirus vaccination [16,20]. An age-structured cohort model was developed in Excel applying a time horizon of five years with time cycles of one month for children less than one year of age and annual thereafter [21-23]. Outcomes in the model were classified by severity and included home-treated community-acquired diarrhea and rotavirus infection leading to GP consultations and/or hospital admissions (including emergency department visits), nosocomial infections and death. Specific characteristics of the model are the ability to take waning immunity, maternal protection against infection through breastfeeding, and herd protection into account. However, the model is not a transmission dynamic model because herd protection is incorporated by straightforward calculus only, using a static approximation based on a fixed fraction of the direct effects. In the base-case analysis for the Netherlands, QALY losses of caregivers were not included and the QALY decrement for children was based on a combination of two published studies performed in Canada and the United Kingdom [24,25]. Similar to the Roxanne-model CoRoVa also used the At Risk software for (probabilistic) sensitivity analyses.

### Standardized input parameters

For this study, analyses of the models were provided regarding their structure and outcomes for four hypothetical countries, representative of different continents and income levels (low, middle or high), respectively classified into the WHO geographical regions and mortality strata [14]. In particular, sets of standardized input parameters were provided to the modellers and analyzed for a hypothetical Afr E country representing the African region with high child and very high infant mortality; a hypothetical Sear D country representing the South-east Asian region with high child and adult mortality; a hypothetical Amr D country representing the South and Middle American regions with again high child and adult mortality; and a hypothetical Eur A country representing developed countries in the European setting. Tables 2, 3, 4 and 5 summarize the set of standardized input parameters. Rectangular age distributions were assumed, implying that life expectancy decreases with one year exactly for every one-year increase in the age of infants and toddlers considered. (For example, if life expectancy is 70 at birth, it would be 69 at the age of 1, 68 at the age of 2 and so on). Simplifying assumptions were justified as our interest concerned the comparison between models rather than the exact representation of country-specific demographic, epidemiological and economic impacts.

**Table 2:** Standardized dataset for the cost-effectiveness models in rotavirus vaccination: demography and incidence (Sources: [4,5,7,14,15,20,26,27])

	Afr	Sear	Amr	Eur	Notes
Total # of life births	1,496,200	3,427,800	140,110	190,000	
Life expectancy at birth in years	54	66	73	80	average men & women
Population	34,255,722	141,822,276	5,486,685	16,500,000	
% of population < 5 years	16.8%	16.8%	16.8%	6.1%	
% urban	42%	23%	62%	100%	
Infant mortality (< 1 year of age)	64	45	21	4	per 1000 life births
Mortality < 5 years	104	57	26	0.5	per 1000 life births/yr
Population < 5 years	5,736,373	17,399,197	730,913	1,000,000	
Incidence mild rotavirus gastro-enteritis					
1st year after birth	1.4%	1.4%	1.4%	1.4%	in % per month
2nd year after birth	0.57%	0.57%	0.57%	0.57%	in % per month
3rd & 4th after birth	0.49%	0.49%	0.49%	0.49%	in % per month
Incidence of moderate	38.7%	38.7%	38.7%	33.1%	in % from mild
Incidence of severe	7.9%	7.9%	7.9%	12.1%	in % from moderate
Incidence of death	18.8%	12.5%	6.3%	0.05%	in % from severe
Incidence of nosocomial infections	33.3%	33.3%	33.3%	25%	% from severe (on top)

**Table 3:** Standardized dataset for the cost-effectiveness models in rotavirus vaccination: vaccine characteristics (Sources: [1-5,7,15,20,28], expert opinions)

	Afr	Sear	Amr	Eur	Notes
Efficacy, assuming a 2-dose schedule at 2 & 3 months (1 dose only between brackets)					
Mild	52% (52%)	52% (52%)	52% (52%)	87% (87%)	
Moderate	55% (54%)	55% (54%)	55% (54%)	92% (90%)	
Severe	60% (54%)	60% (54%)	60% (54%)	100% (90%)	
Waning of efficacy (annual)					
Mild & moderate	0.63	0.63	0.63	0.63	multiply each next year
Severe	0.83	0.83	0.83	0.83	multiply each next year
Coverage					
Dose 1	50%	50%	50%	50%	
Dose 2	40%	40%	40%	40%	
20 years after introduction	80%	80%	80%	96%	for both doses
Coverage improvement	linear	linear	linear	linear	
Per-dose vaccine costs (2 doses)					
2009-2014	7.5	7.5	7.5	45	
2015 & beyond	4	4	4	45	

**Table 4:** Standardized dataset for the cost-effectiveness models in rotavirus vaccination: health-care use and costs

	Afr	Sear	Amr	Eur	Notes
Average length of hospital stay	4	4	4	4	days
Cost per hospital day	US$35	US$34	US$122	€ 550	
Cost per outpatient visit (health center/GP)	US$10.50	US$9	US$34.50	€ 40	Community acquired only
Out-of-pocket costs (comm.-acq. only)	US$0.50	US$2.5	US$5	€ 15	diapers/travel/OTC
Total direct costs for nosocomial cases	US$15	US$15	US$50	€ 2,000	
Cost of productivity loss/day	US$1	US$5	US$10	€ 125	
Parents with work loss					
Non-hospitalized	20%	20%	20%	20%	
Hospitalized & nosocomial	75%	75%	75%	75%	
Days of work missed for parents					
Mild	1	1	1	1	
Moderate	1.5	1.5	1.5	1.5	
Severe	2	2	2	2	also for nosocomial
Discount rates	3%	3%	3%	3%	money & health
Administration costs per dose	US$0.53	US$0.46	US$0.46	€ 5	

(Note: all mild cases were treated at home, all moderate additionally in an outpatient setting, such as outpatient hospital, general practitioner or health center and all severe cases additionally in hospital; all cases have out-of-pocket costs), plausible assumptions based on literature [29-31] and expert opinions (MP & MJ))

GP: general practitioner; comm.-acq: community acquired; OTC: over the counter

**Table 5:** Utility losses and some remaining issues [4,5,7,20,27]

	Afr	Sear	Amr	Eur	Notes
Disutility					
Mild	0.15	0.15	0.15	0.15	during 4 days
Moderate	0.25	0.25	0.25	0.25	during 8 days
Severe	0.7	0.7	0.7	0.7	during 11 days
Nosocomial	0.7	0.7	0.7	0.7	during 4 days
Death	1	1	1	1	per year
Age weighting (primarily considered for DALYs)	off	off	off	off	
Perspective	societal & health care	societal & health care	societal & health care	societal & health care	
Herd effect	off	off	off	off	

DALY: disability adjusted life year

Although the time horizon in the single- and multi-cohort models was lifetime, this effectively produced a time horizon of five years after the birth of the last cohort, since it is assumed that no rotavirus gastroenteritis occurs beyond the age of five years and vaccination is only investigated in infants in their first year of life. Various sources were utilized for parameter estimates as indicated in Tables 2, 3, 4 and 5[1-5,7,14,15,20,26-31]. However, as the objective was comparative rather than to exactly mimic the situations of specific countries often plausible assumptions were made rather than exact replications of individual sources. Plausibility of assumptions was primarily based on the expert opinions of two of the authors (MP and MJ).

Key parameters for all analyses were the incidence of rotavirus gastroenteritis (RVGE), the corresponding risks of rotavirus-related health-care use, as well as corresponding costs. All cases with RVGE were assumed to be treated at home. In particular, moderate cases were assumed to involve one additional outpatient visit (GP, health center or hospital) and severe cases were assumed to involve: home treatment, an outpatient visit and a hospitalization. Some models had the additional option of severe cases not being hospitalized. Based on data from the clinical trials, vaccine efficacy could be specified for different outcomes and for high-income versus other countries.

Alternatively to what is expressed in Table 1, incidence could be expressed as average annual risks over the same five years considered in the data and modelling. In particular, the average annual risks at population level are 10,440, 4,040 and 320 per 100,000 people in all developing regions for mild, moderate and severe, respectively. Correspondingly, figures for the Eur region are 10,440; 3,460; and 419 per 100,000 people. Similarly, annual mortality risks are 60, 40, 20 and 0.21 per 100,000 population for the Afr, Sear, Amr and Eur regions, respectively.

### Standardized output

Model developers were requested to present a standardized set of output variables for one single cohort (for the multi-cohort model, the coordinating center estimated the results for one cohort themselves). In particular, these were: total number of persons and (person-years if available) followed in the model; undiscounted number of mild cases of RVGE; undiscounted number of moderate cases of RVGE; undiscounted number of severe cases of RVGE; undiscounted number of outpatient visits (GP, outpatient or health center; typically this would equal the number of moderate and severe cases); undiscounted number of hospitalizations (typically equal to the number of severe cases); undiscounted number of nosocomial cases (equal to 1/4 or 1/3 of the number of hospitalizations, in the Eur and other regions, respectively); undiscounted number of deaths; discounted direct outpatient costs; discounted direct inpatient costs; discounted vaccination costs; discounted and undiscounted QALYs due to deaths (difference between vaccination and no vaccination is equal to the number of life-years gained); discounted and undiscounted QALYs due to morbidity; cost/QALY from the health-care perspective; cost/QALY from the societal perspective; and finally, a sensitivity analysis was requested by varying parameters values through halving and doubling their base case values, except the discount rate which was investigated for alternative values of 0% and 4%.

In particular, cost-effectiveness was expressed in net costs per QALY gained by subtracting discounted savings from the reduced need for RVGE treatments from (discounted in the multi-cohort model) vaccination costs to provide the numerator and dividing by the QALYs gained (the denominator).

## Results

Results of using the standardized dataset for the various regions in different models are shown in Tables 6, 7, 8, 9 and 10 for all three models. In general, cost-effectiveness results are broadly similar and comparable.

**Table 6:** Comparative analysis on costs per QALY

	POLYMOD	Roxanne	CoRoVa
Health-care perspective			
Afr	$ 265a	$ 188-367^b^	$ 233-440 ^b^
Sear	$ 358a	$ 257-503^b^	$ 308-591 ^b^
Amr	$ 307a	$ 200-652^b^	$ 336-862 ^b^
Eur	€ 57,897	€ 50,999	€ 56,656
Societal perspective			
Afr	$ 260a	$ 185-364 ^b^	$ 231-438 ^b^
Sear	$ 328a	$ 241-487 ^b^	$ 293-577 ^b^
Amr	$ 196a	$ 143-595 ^b^	$ 282-809 ^b^
Eur	€ 49,427	€ 40,041	€ 44,263

a. based on future reduction of vaccine prices from $7.5 to $4; b. Range given for previous upper and lower vaccine prices

**Table 7:** Outcomes predicted by models for one birth cohort in Afr-region

	POLYMOD^a^	Roxanne	CoRoVa
Undiscounted cases			
Mild	92,989	121,312	128,807
Moderate	49,051	70,126	54,559
Severe	5,092	9,589	5,277
Nosocomial	1,680	1,986	2,701
Deaths	955	1,789	1,524
Outpatient	54,143	70,126	54,559
Inpatient (comm. acq.)	5,092	9,589	5,277
Discounted savings			
Outpatient	$568,502	$716,305	$623,977
Inpatient	$973,247	$1,329,538	$753,884
Indirect	$108,916	$106,377	$81,486
Discounted net costs^b ^(*1000)	$6,759	$ 8,757-17,094	$ 9,250-17,467
Discounted QALYs			
Mortality	24,962	45,817	39,070
Morbidity	542	762	628

a. Approximation for one cohort from the multi-cohort results; b. Health-care perspective and range given for previous upper and lower vaccine prices if appropriate.

**Table 8:** Outcomes predicted by models for one birth cohort in Sear-region

	POLYMOD^a^	Roxanne	CoRoVa
Undiscounted cases			
Mild	216,205	276,861	299,907
Moderate	114,255	159,129	127,020
Severe	11,887	22,086	12,325
Nosocomial	3,923	4,748	6,309
Deaths	1,486	2,752	2,366
Outpatient	126,142	159,129	127,020
Inpatient (comm. acq.)	11,887	22,086	12,325
Discounted savings			
Outpatient	$1,135,274	$1,391,130	$1,852,744
Inpatient	$2,208,959	$2,975,397	$1,712,409
Indirect	$1,267,227	$1,213,598	$948,564
Discounted net costs^b ^(*1000)	$15,424	$ 19,969-39,083	$ 20,545-39,423
Discounted QALYs			
Mortality	41,822	75,969	65,244
Morbidity	1,261	1,731	1,462

a. Approximations for one cohort from the multi-cohort results; b. Health-care perspective and range given for previous upper and lower vaccine prices if appropriate.

**Table 9:** Outcomes predicted by models for one birth cohort in Amr- region

	POLYMOD^a^	Roxanne	CoRoVa
Undiscounted cases			
Mild	8,989	11,318	12,481
Moderate	4,760	6,471	5,286
Severe	496	897	514
Nosocomial	164	193	263
Deaths	31	59	50
Outpatient	5,256	6,471	5,286
Inpatient (comm. acq.)	496	897	514
Discounted savings			
Outpatient	$181,333	$216,626	$239,534
Inpatient	$330,083	$432,618	$255,186
Indirect	$105,467	$98,697	$78,947
Discounted net costs^b ^(*1000)	$293	$ 346-1,127	$ 495-1271
Discounted QALYs			
Mortality	900	1,662	1,413
Morbidity	53	66	61

a. Approximations for one cohort from the multi-cohort results; b. Health-care perspective and range given for previous upper and lower vaccine prices if appropriate

**Table 10:** Outcomes predicted by models for one birth cohort in Eur-region

	POLYMOD^a^	Roxanne	CoRoVa
Undiscounted cases			
Mild	22,708	32,186	28,680
Moderate	10,360	14,580	10,430
Severe	1,658	2,324	1,806
Nosocomial	415	528	452
Deaths	1	1	1
GP-visits	12,019	14,580	10,430
Inpatient (comm. acq.)	1,658	2,324	1,860
Discounted savings			
Outpatient	€480,745	€556,984	€829,111
Inpatient	€4,643,286	€5,949,119	€4,714,920
Indirect	€ 1,322,021	€ 2,111,857	€ 2,088,993
Discounted net costs ^b^	€ 9,032,932	€ 9,842,807	€ 9,518,208
Discounted QALYs			
Mortality	25	27	26
Morbidity	131	166	142

a. Approximations for one cohort from the multi-cohort results; b. Health-care perspective.

However, there are differences in the building blocks of these cost-effectiveness ratios, for example, regarding estimations of QALY losses related to morbidity in the three models. The results for the CoRoVa model were generally in between those for the other two. The Roxanne model generally predicts an overall higher number of cases, most notably for hospitalized cases. This translates into relatively high inpatient savings that are however lowered by discounting and overall dominated by vaccination costs to render similar net discounted costs for all three models investigated. However, the POLYMOD model gave consistently lower results for these discounted net costs; this might be due to the approximation from results for the multiple cohorts to just one. Also, higher vaccination costs in later years in the multi-cohort model were offset by a higher number of cases averted in the single-cohort model, resulting in the comparable cost-effectiveness ratios between the two types of models. Results suggest that rotavirus vaccination could potentially be cost-effective in all regions, particularly in low and middle income countries. However, the standardized data set is highly generalized and not specific to any individual country, so conclusions to support policy making should not be drawn in the absence of country-specific analyses.

**Figure 1:**
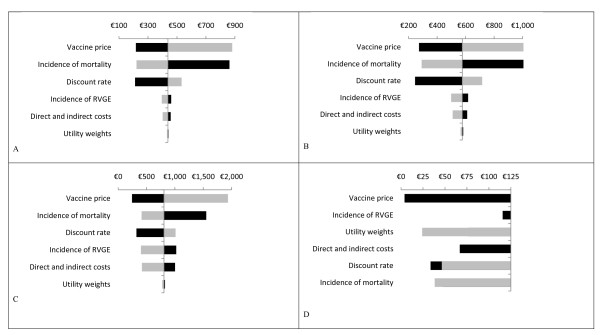
Sensitivity analyses on the base case cost effectiveness ratio ($ per QALY, except in D where € 's *1000 were used) using the CoRoVa model for Afr (A), Sear (B), Amr (D), and Eur (D) regions. Parameters were varied through halving and doubling, except for the discount rates which were 0% and 4% for both costs and health effects. Dark bars show the incremental cost effectiveness ratio after a 100% decrease in the parameter, whereas light bars show the incremental cost effectiveness ratio after a 100% increase (note that an increase in the incidence or the costs resulted in negative cost effectiveness ratios in the Eur region [D]). Note that when the incidence of RVGE was increased or decreased, the total number of deaths was kept constant to identify the sole effect of incidence. QALY, quality adjusted life year; RVGE, rotavirus gastroenteritis.

Figure 1 shows the sensitivity analysis performed on the model results, with the example of CoRoVa shown here (other models showed similar patterns). From previous work on cost-effectiveness of rotavirus vaccination [5,7,15,19], it is well-known that generally these results are highly sensitive to the vaccine price, rotavirus-associated mortality and the discount rate. Our analysis shows a similar pattern for all regions investigated. However, there is an interesting shift observed in variables influencing the cost-effectiveness results between EU and non-EU countries. In the EU region the incidence of the disease is a driver followed by utility weights whereas in the non-EU region mortality is essentially driving the result.

## Discussion

We identified various models used to estimate the cost-effectiveness of rotavirus vaccination. From these, results using the standardized dataset could be obtained for three specific applications (the POLYMOD, Roxanne and CoRoVa models). Despite differences in the approaches, cost-effectiveness results of the models were quite similar. Differences between the outcomes of the specific building blocks of the cost-effectiveness (that is, vaccination costs, savings and QALYs gained) of the models currently investigated seem to relate to five aspects of the models: the multi-cohort nature of the POLYMOD model which assumed a step up in vaccination coverage (and hence in vaccination costs as well as cases prevented); the exact timing of the waning in the models and in particular the exact modulation of between-dose efficacies in the CoRoVa model; assumptions about the distribution of cases of different severity levels within the one-year age groups provided (for example, assuming a Weibull distribution in the Roxanne model); and the possibility in the models of experiencing subsequent episodes of rotavirus infections and/or experiencing episodes with multiple manifestations (for example, first moderate progressing to severe) and types of health-care use (for example, inpatient and outpatient, rather than just one of both).

Ergo, differences between the models on the individual components of cost-effectiveness could be related to some specific generic features of the models with regards to representing vaccine uptake and pricing, within age-group distributions, waning and between-dose efficacies and inclusion of additional groups and episodes in the general design of these models. Sensitivity analysis revealed that cost-effectiveness of rotavirus vaccination is highly sensitive to vaccine prices, rotavirus-associated mortality and discount rates, in particular that for effects. This is fully in line with other authors' findings [32].

Unfortunately, we were not able to include a model with a transmission dynamic approach in our model comparison, instead of the cohort approach followed in the three models analyzed. Emerging evidence that herd immunity effects might be relevant for rotavirus transmission and vaccination enhances the relevance of considering populations and transmissions between cohorts [33-35]. Inability to include these models was often related to the complexity of these models and the difficulties to adequately grasp these complexities in the standardized framework provided on inputs and outputs. However, for further work it is important to also analyze such dynamic models given their major advantage of incorporating infection dynamics including herd immunity effects and potential age shifts in epidemiology [36]. Also, differences in uptake between high-, middle-, and low-income countries should be analyzed using dynamic models given the different impacts of coverage levels on herd immunity.

The three models selected for our analysis were all basically developed for high-income countries. For reasons stated previously, other models - inclusive of models developed initially for low- and middle-income settings - could unfortunately not be considered in our comparison. As one consequence, all the publications arising from these models involved costs per QALY gained rather than costs per DALY Disability Adjusted Life Year averted. Although not undisputed, for low- and middle-income settings DALYs rather than QALYs are the common metric used [27,37]. We do note that one specific study showed that results differ only slightly if DALYs are used instead of QALYs; in particular, it showed slightly more favorable cost-effectiveness for DALYs as the outcome [16]. However, it has been demonstrated that the decision about whether or not to include caregiver QALYs has a major impact on results [4,7]. The appropriateness of including QALYs beyond the index case of disease is being debated; it could be argued that caregivers' QALYs are particularly important for rotavirus as they can be measured directly, and hence may be more valid than QALYs in small children with RVGE, where proxy measurements have to be used [7,21,22].

Our findings for the regions Afr, Sear, Amr and Eur should not be considered as exact representative results and policy making should not directly be based on this. For example, it is very unlikely that the similarities assumed for the proportion of RVGE cases that are mild, moderate and severe are valid in real world. This simplifying assumption was made in order to test the models generically and consistently. Nevertheless, our results clearly indicate a general trend of increasingly more favorable cost-effectiveness when going from high- to middle- and on to low-income countries, respectively. As the sensitivity analysis shows, this is obviously primarily related to vaccine pricing and the QALY-impact of averted mortality due to rotavirus infection. However for actual policy making, countries will need to either further consider the results from existing studies and reviews, or initiate country-specific cost-effectiveness analyses. For countries that have the capacity and resources to model the cost-effectiveness of rotavirus vaccine, our comparative analysis can help inform the design of new models or selection of existing models to support national-level decision making.

Hence, although our analysis is not meant to directly inform policy making, it offers considerable guidance for design and/or selection of a model for adaptation to individual (low-income) countries that want to conduct cost-effectiveness analyses. Scarce resources in these countries may direct the choice towards adapting an existing model rather than initiating the development of a new approach. Reassuringly, our analysis suggests that different models produce similar cost-effectiveness estimates, illustrating that the exact choice of which model to adapt may not be as crucial as the choice of assumptions and parameter values to incorporate in the model.

## Conclusions

We conclude that our approach is helpful on two specific levels. Firstly, the comparative approach followed here is helpful in understanding the various models selected and will thus benefit (low-income) countries in designing their own cost-effectiveness analyses using new or adapted existing models. Secondly, we find that the communication between the individual researchers involved in the three models was helpful in the further development of these individual models and will be so in the future. Therefore, we recommend that this kind of comparative study be extended to other areas of vaccination and even other infectious disease interventions, beyond the three areas that have been explored by WHO (pneumococcal vaccination, human papilloma virus vaccines and (here) rotavirus) [38-40].

Finally, the models reviewed in the exercise gave similar and comparable results which appear to have face validity. Hence it appears possible to recommend their use in policy settings, at least for high income countries. However, potential users of the models need to consider the specific building blocks of the cost-effectiveness models including the nature, scope, design and assumptions made and how they affect outcomes. Potential users of the models in low and middle income countries need to consider results from existing studies and reviews. There will be a need for contextualization including the use of country specific data inputs. However, given that the underlying biological and epidemiological mechanisms do not change between countries, users are likely to be able to adapt existing model designs rather than developing completely new approaches. Also, transmission dynamic effects are likely to be important, particularly when considering the effect of vaccination (since vaccination can affect other cohorts besides those vaccinated). Hence, we would recommend that future cost-effectiveness tool comparison exercises include dynamic models.

## List of abbreviations

CoRoVA: Consensus Model on Rotavirus Vaccination; ER: emergency room; QALY: disability adjusted life year; DALY: disability adjusted life year; Roxanne: Rotarix™ Analyses of Economics; RVGE: rotavirus gastroenteritis; WHO: World Health Organization; Afr: African; Eur: European; Sear: South-East Asian; Amr: Americas.

## Competing interests

MP received a grant from SPMSD to perform research into the cost-effectiveness of rotavirus vaccination in the Netherlands, within the framework of MR his PhD-work. The grant was fully unrestricted and as such accepted by the Dutch Health Council to contribute to the advice on whether to integrate rotavirus vaccination in the Dutch National Immunization Program (discussion still ongoing, as of February 28^th ^2011). BS is an employee of GlaxoSmithKline Biologicals in Wavre (Belgium). All the other authors declare that they have no competing interests.

## Authors' contributions

MP & RH conceived the study. MP, MJ, BS, MR and HT integrated the results from different models and drafted the manuscript with input from the other authors. All authors provided advice on the methodology and the data analyses of tool comparison exercise. All authors read and approved the final manuscript.

## Pre-publication history

The pre-publication history for this paper can be accessed here:

http://www.biomedcentral.com/1741-7015/9/84/prepub
